# Amelanism in the corn snake is associated with the insertion of an LTR-retrotransposon in the *OCA2* gene

**DOI:** 10.1038/srep17118

**Published:** 2015-11-24

**Authors:** Suzanne V. Saenko, Sangeet Lamichhaney, Alvaro Martinez Barrio, Nima Rafati, Leif Andersson, Michel C. Milinkovitch

**Affiliations:** 1 Laboratory of Artificial & Natural Evolution (LANE), Department of Genetics & Evolution, University of Geneva, Switzerland; 2 Science of Life Laboratory Uppsala, Department of Medical Biochemistry and Microbiology, Uppsala University, Uppsala, Sweden; 3 Department of Animal Breeding and Genetics, Swedish University of Agricultural Sciences, Uppsala, Sweden; 4 Department of Veterinary Integrative Biosciences, College of Veterinary Medicine and Biomedical Sciences, Texas A&M University, College Station, USA; 5 SIB Swiss Institute of Bioinformatics, Geneva, Switzerland

## Abstract

The corn snake (*Pantherophis guttatus*) is a new model species particularly appropriate for investigating the processes generating colours in reptiles because numerous colour and pattern mutants have been isolated in the last five decades. Using our captive-bred colony of corn snakes, transcriptomic and genomic next-generation sequencing, exome assembly, and genotyping of SNPs in multiple families, we delimit the genomic interval bearing the causal mutation of amelanism, the oldest colour variant observed in that species. Proceeding with sequencing the candidate gene *OCA2* in the uncovered genomic interval, we identify that the insertion of an LTR-retrotransposon in its 11^th^ intron results in a considerable truncation of the p protein and likely constitutes the causal mutation of amelanism in corn snakes. As amelanistic snakes exhibit white, instead of black, borders around an otherwise normal pattern of dorsal orange saddles and lateral blotches, our results indicate that melanocytes lacking melanin are able to participate to the normal patterning of other colours in the skin. In combination with research in the zebrafish, this work opens the perspective of using corn snake colour and pattern variants to investigate the generative processes of skin colour patterning shared among major vertebrate lineages.

Understanding the determinism of trait diversification is a key theme in evolutionary biology in general, and evolutionary developmental biology (EvoDevo) in particular. Vertebrate skin coloration provides an effective model system for exploring the genetic determinism and developmental plasticity associated with phenotypic variation. Squamates (lizards and snakes) exhibit a remarkable set of pigmentary and structural elements generating a broad variety of colours and colour patterns (*e.g.*^1^,^2^,^3^,^4^,^5^,^6^,^7^). Colour polymorphisms in squamates have been used as examples of adaptive evolution associated with habitat, behaviour, reproductive strategies, ageing, immune response, and speciation^8^,^9^,^10^,^11^.

We are promoting the corn snake (*Pantherophis guttatus*), a Colubridae species from the southestern United States of America, as an ideal snake model for evolutionary developmental studies^12^,^13^,^14^,^15^. Indeed, corn snakes are oviparous, harmless and sufficiently small (generally <1.5 m) to be easily maintained and bred in captivity, and they exhibit multiple colour morphs, enabling systematic analysis of the molecular pathways controlling adaptive colour (and colour pattern) variation in reptiles.

As in many other lineages^16^,^17^,^18^,^19^,^20^,^21^,^22^,^23^, melanin-based coloration in squamates is highly variable and likely plays crucial roles in thermoregulation, camouflage, UV protection, and sexual selection (e.g.^24^,^25^,^26^). Amelanism is the oldest colour trait variant to have been noticed in corn snakes^27^ and it has been observed in Florida, North Carolina and Tennessee^28^. Amelanism is expected to be genetically heterogenous and may reflect mutations at different loci with a key role in melanogenesis as well as different rare mutations at the individual loci. Amelanistic corn snakes exhibit a normal pattern of orange dorsal saddles and lateral blotches (made of uncharacterised pigments), but the black borders around these marks are replaced with white skin (Fig. 1). As they entirely lack black pigment, amelanistic corn snakes also exhibit pink eyes and do not show the characteristic black checkers on their ventral scales (Fig. 1). In 1953, one amelanistic snake from North Carolina was incorporated into a small captive-breeding population and subsequent crosses demonstrated that the trait is determined by a single-locus recessive mutation^28^,^29^,^30^. Today, the amelanistic morph is the most frequent variant in captive-bred populations across the world, and all individuals exhibiting that trait are thought to bear the mutation of the founding North Carolina individual.

In vertebrates, melanin is synthesised from the amino-acid tyrosine in vesicular organelles (melanosomes) of neural-crest derived cells called melanophores (or melanocytes) that interact with the endocrine, immune, and nervous systems^31^,^32^. A substantial number of genes whose mutations are associated with defects in melanin-based pigmentation have been identified in humans and model species^33^,^34^,^35^,^36^,^37^,^38^,^39^,^40^,^41^. Investigation of the molecular genetic determinism of such colour variants in squamates is hindered by the lack of extensive genomic resources and has thus so-far mainly relied on sequencing candidate genes, an approach that has been successful in some cases (e.g.^42^) but ineffective in many others (e.g.^43^,^44^,^45^).

Positional cloning of mutations determining traits of interest is a more effective method which has been successfully performed for many years in model and domestic species^46^. This procedure is however complex as it requires both controlled pedigrees (to generate a recombinant mapping population) and a dense and large set of genetic markers (*e.g.*, microsatellites or Single Nucleotide Polymorphisms, SNPs) that co-segregate with the phenotype of interest, hence allowing the identification of the genomic interval harbouring the causal mutation. The so-called ‘next generation sequencing’ (NGS) methods have opened the possibility to perform mapping-by-sequencing (*i.e.*, the simultaneous mapping and identification of causal mutations), in species with excellent genomic resources^47^, but also allows performing efficient linkage mapping or genome-wide association analysis in species with no prior genomic sequence available^48^.

Here, using our captive-bred colony of corn snakes, we combine an unbiased NGS and exome assembly approach^48^, extensive genotyping and candidate gene sequencing, and we identify the insertion of a retrotransposon in the *OCA2* gene as the causal mutation determining the amelanistic phenotype in corn snakes.

## Results

### NGS and exome assembly delineate the genomic interval for the *amel* locus

First, we generated a mapping panel consisting of seven families, with a total of 140 wild-type (WT) and 116 amelanistic progeny, by crossing homozygous amelanistic snakes with WT individuals heterozygous for the *amel* allele (see Methods). Second, we *de-novo* sequenced the corn snake transcriptome using normalised cDNA prepared from multiple organs and developmental stages, generating 2.7–3.2 Gbp of high-quality (Q ≥ 30) data. The corresponding 275 million paired-end reads were assembled (see Methods) into a non-redundant transcriptome containing 92,308 contigs with a cumulative length of 35.7 Mbp (N50 = 423 bp). Third, we selected one of the seven families and sequenced (9.5–19.4x coverage) four genomic DNA libraries generated with samples from *(i)* the heterozygous mother (*amel*/+ genotype), *(ii)* the amelanistic father (*amel*/*amel* genotype), *(iii)* 20 WT heterozygous offspring individuals and *(iv)* 20 amelanistic offspring individuals.

We then used the genomic reads of the father to extend exons of the cDNA contigs with intronic and intergenic sequences, in a process called exome assembly^48^. This strategy provides better alignability of genomic reads at exon/intron boundaries and improves SNP calling. We used this exome assembly as a reference to separately align genomic reads and call SNPs in the four DNA samples (*i.e.*, father, mother, and two pools of offspring). After filtering the 510,724 reported variants (see Methods), further analyses were performed only with the 3,278 SNPs (in 2,186 exome contigs) that met the quality and coverage criteria as well as perfectly matched the expected allelic distribution at the amelanistic locus (*i.e.*, heterozygous in the WT mother and offspring, and homozygous in the amelanistic father and offspring). Contigs with two or more SNPs that did not perfectly co-segregate were then identified as false positives and the 1,025 remaining contigs were aligned (tBLASTx) against the genome assembly of the green Carolina anole lizard (*Anolis carolinensis*) AnoCar2.0^49^, the only squamate species with a genome sequence assembled into chromosomes. Among the 368 corn snake contigs with a hit (*i.e.*, with an ortholog in the *A. carolinensis* genome), 146 match the anole lizard chromosome 3, of which a vast majority (103 contigs) define an interval between coordinates 82.7 and 119.5 Mbp. Given the relatively high conservation of synteny between lizard and snake genomes (e.g.^50^), this strategy is likely to have identified the anole lizard genome interval that is orthologous to the corn snake genome region including the *amel* locus. Analysis of the draft corn snake genome confirms this interval^15^.

### Reducing the genomic interval and identifying candidate genes

**Figure 2:**
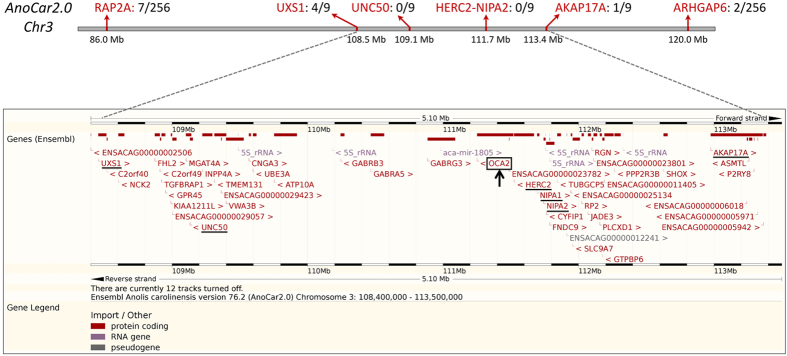
Linkage mapping of the *amel* locus; NGS and exome assembly delineate the genomic interval for the *amel* locus. Top: Starting from an exome assembly, the identification of SNPs co-segregating with the amelanistic mutation delineates an orthologous genomic interval of 4.9 Mbp in the anole lizard genome AnoCar2.0^49^; numbers above the line indicate the number of recombinants for genetic markers in seven genes (name in red). Bottom: physical map of the interval (Ensembl genome browser; http://www.ensembl.org/) defined by markers in the genes *uxs1* (left, 4 recombination events) and *akap17a* (right, 1 recombination); the gene *OCA2* (black arrow and frame) is the best candidate for bearing the amelanistic mutation.

To confirm the results of the initial mapping performed on a single family, we genotyped (by sequencing) SNPs in two genes flanking the identified interval (*RAP2A*, Chr3:86.073–86.099 Mbp and *ARHGAP6*, Chr3:119.453–119.496 Mbp) in all 256 offspring of the seven families segregating the trait (Fig. 2, Supplementary Table S1). Many of the SNPs in this interval showed no recombination with the *amel* locus providing conclusive evidence that it is located in this interval; this corresponds to a lod score >74.2 (a value of 3 is usually considered significant). We identified seven recombinants between the *amel* locus and SNPs in the *RAP2A* gene and two recombinants between *amel* and SNPs in the *ARHGAP6* gene. We then used these nine individuals to reduce the genomic interval of interest by genotyping SNPs in five additional genes. We identified four and one recombination events between the *amel* locus and *UXS1* (Chr3:108.455–108.515 Mbp) and *AKAP17A* (Chr3:112.976–113.359 Mbp), respectively, whereas no recombination was observed for SNPs in *UNC50*, *HERC2*, and *NIPA2* (Fig. 2, Supplementary Table S1). Hence, the interval containing the *amel* locus in the genome of *A. carolinensis* is located between 108.5 and 113.4 Mbp on chromosome 3.

Even though this 4.9 Mbp interval is relatively large and contains 46 protein coding genes, one of these, the *oculocutaneous locus 2* (*OCA2*) is an excellent candidate for the *amel* locus. Indeed, structural and regulatory variations of the *OCA2* gene seem to be major determinants of human skin, hair and eye colour^51^ and can cause oculo-cutaneous albinism type II in mouse and human^52^,^53^ as well as in *Astyanax* cavefish^22^. OCA2 is a 12-transmembrane domain protein that localises at the membranes of melanosomes where it likely acts as an ion-transporter and plays a substantial role in the establishment or maintenance of the melanosomal acidic pH required for proper tyrosinase activity^54^,^55^,^56^,^57^,^58^.

### An LTR-retrotransposon insertion generates a loss-of-function *OCA2* in corn snakes

**Figure 3:**
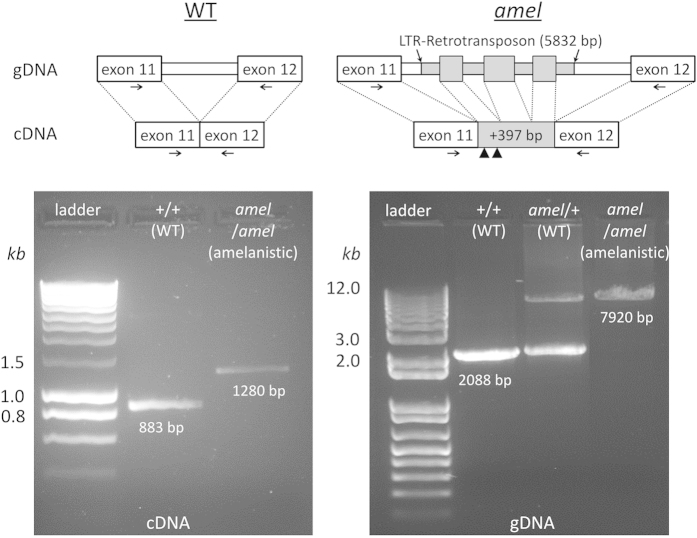
Diagram of genomic DNA (gDNA) and corresponding cDNA organisation of the *OCA2* junction between exons 11 and 12 of the wild type (WT) and amelanistic (*amel*) alleles. Top: The *amel* mutation corresponds to the insertion of a 5,832-bp fragment (grey) into the intron 11–12 of the *OCA2* gene. Three fragments (not drawn to scale) of this insert are spliced together as an additional 397-bp sequence which contains two premature stop codons (arrowheads) at its 5′ end. Bottom: gel images of PCR products of the *OCA2* fragments from cDNA (left) or gDNA (right) of WT and amelanistic individuals. The positions of PCR primers (horizontal arrows in the top diagram) are approximate and are actually different for cDNA and gDNA.

Using cDNA prepared from skin samples of four homozygous WT (+/+) and four amelanistic (*amel*/*amel*) corn snake individuals, we amplified and sequenced 2,776-bp containing the full-length *OCA2* transcript. The sequence included a full open reading frame of 2,532-bp corresponding to a 843-aa protein (one aa shorter than in the anole lizard) and a stop codon. The amplification of the orthologous cDNA fragment from four amelanistic corn snake individuals generated the same sequence but with an additional 397-bp fragment inserted between exons 11 and 12 (Fig. 3; Supplementary Data S1). This insertion contains two stop codons and, hence, generates a considerably truncated variant (402 aa instead of 843 aa) of the OCA2 protein which is therefore very likely to be non-functional.

To uncover the origin of the 397-bp insertion, we used primers in exons 11 and 12 and amplified by long-range PCR the connecting intron from genomic DNA samples of homozygous WT (+/+), amelanistic (*amel*/*amel*) and heterozygous (*amel*/+) corn snakes. Using sequencing by primer walking, we found that the intron is 2,011 bp in the wild type allele but contains a 5,832-bp insertion (total length of intron = 7,843 bp) in the *amel* allele (Supplementary Data S2). Comparing the 5,832-bp genomic DNA insertion to the 397-bp cDNA insertion, we identified that the latter is formed by splicing of three fragments of the former. Each of these three new exons are flanked by the expected 5′ GT and 3′ AG splice sites.

We aligned the 5,832-bp genomic fragment against a reference collection of repeats from the Repbase database^59^ and obtained a hit with the Copia-6 LTR retrotransposon of *A. carolinensis* (CENSOR score 1,766, similarity 0.399). Moreover, we searched for conserved domains on the NCBI database (http://www.ncbi.nlm.nih.gov/Structure/cdd/wrpsb.cgi) and identified the RNase H domain of the Ty1/Copia family (351–770 bp, e-value 4.18^−46^), a reverse transcriptase (1,029–17,51 bp, e-value 1.64^−80^), an integrase core domain (2,490–2,837 bp, e-value 3.08^−24^), and a gag-polypeptide of the LTR-copia type (3,828–4,160 bp, e-value 1.08^−07^), all indicative of the presence of a retrotransposon.

## Discussion

Non-mammalian vertebrates exhibit a broad range of colours generated through interactions between pigmentary chromatophores and structural nanoscopic elements (such as arrays of guanine nanocrystals producing light interference within iridophore cells)^1^,^2^,^3^,^4^,^5^,^6^,^7^. In lizards for example, we have shown that the final colour of a given patch of skin is strongly influenced by at least three broadly-defined interacting parameters^4^,^7^: *(i)* the nature of the pigments in pigmentary cells such as xanthophores and erythrophores, *(ii)* the pH or redox state of these pigments, and *(iii)* the geometric parameters of the structural elements. The latter includes the size, shape and orientation of the iridophore nanocrystals as well as the geometry and level of order of the lattice of these elements. Depending on the species, position on the body, as well as the physiological and behavioural state of the animal, these interactions can produce various colours in the visible range. Chameleons have even the remarkable ability to exhibit complex and rapid structural colour changes through active tuning of a lattice of guanine nanocrystals within a superficial thick layer of dermal iridophores^7^.

It remains a major challenge to investigate in non-mammalian vertebrates the behaviour of various cell types (pigmentary and structural), and their interactions during development and homeostasis, so as to understand the final skin colour and colour patterns^60^,^61^,^62^,^63^,^64^. For example, several species of tropical day geckos of the genus *Phelsuma* exhibit a precise colocalisation of red chromatophores with underlying structural disorganised broadband reflectors (*i.e.*, iridophores characterised by a disordered lattice of nanocrystals), whereas the rest of the dorsolateral skin combines yellow chromatophores with highly ordered narrowband (generally blue/green) reflectors. The availability of many mutants, as well as recent transgenic and imaging tools, is making the zebrafish (*Danio rerio*) an ideal model to study this kind of cellular interactions during development^63^. However, if one wants to extract general principles for vertebrate colour and colour pattern formation, we think it is important to additionally investigate these traits in species of other major vertebrate lineages.

We have recently promoted the corn snake *Pantherophis guttatus* as particularly appropriate for evolutionary developmental studies^12^,^13^,^14^ because of multiple practical parameters: corn snakes are oviparous, easy to breed, and harmless (small, non-venomous, and reluctant to bite). The corn snake is also particularly pertinent for investigating the processes generating colours in reptiles because numerous colour and pattern mutants have been isolated in the last 50 years, mostly in private captive-bred colonies. In addition, an annotated draft genome^15^ and an extensive multi-organ and multi-developmental-stage annotated transcriptome (www.reptilian-transcriptomes.org)^65^,^66^ of the corn snake have been recently published for facilitating comparative genomics/transcriptomics, differential expression analyses, as well as developmental and evolutionary studies. Note that this draft genome allows to identify genomic interval harbouring traits of interest (such as the amelanitic locus) with a similar efficiency as the exon assembly approach^15^.

It is indisputable that the very large variation of colours and colour patterns in reptiles (especially squamates, *i.e.*, lizards and snakes) is essentially due to the presence of multiple pigments and structural elements, whereas mammals exhibit only black/brown melanins and essentially no structural colour. However, the importance of melanin in squamates should not be underestimated. For example, many squamates can rapidly disperse/aggregate melanin-containing melanosomes within dermal melanophores to modify skin brightness (without changing much the skin hue) for camouflage, communication and thermoregulation (e.g.^67^,^68^,^69^). Because of its broadband absorbance, melanin can sometimes dominate colour patterns even in the presence of other pigments or of structural colours. Finally, multiple studies have shown the importance of melanocytes in the interactions with other cell types for the development and maintenance of colour patterns in the zebrafish^61^,^63^,^70^,^71^.

Here, using next-generation sequencing of transcriptomic and genomic libraries, exome assembly, and genotyping of SNPs in multiple families of corn snakes, we delimit the genomic interval bearing the causal mutation of the amelanistic trait in corn snakes. Proceeding with sequencing candidate genes in the uncovered genomic interval, we identify that the insertion of an LTR-retrotransposon in an intron of the *OCA2* gene results in a truncated form of the p protein and likely constitutes the causal mutation of amelanism, *i.e.*, the oldest colour trait variant observed in this species^27^. As amelanistic corn snakes exhibit white, instead of black, borders around an otherwise normal pattern of dorsal orange saddles and lateral blotches, our results indicate that melanocytes lacking melanin are still able to participate to the elaboration of a normal patterning of the snake skin. Using a similar approach with the set of available pattern mutants of the corn snake, we anticipate to identify the corresponding causal mutations that would then inform us on the generative processes of skin colour patterning possibly shared among major lineages of vertebrates.

## Materials and Methods

### Animals and ethics statement

Maintenance of, and experiments on animals were approved by the Geneva Canton ethical regulation authority (authorisation GE/82/14) and performed according to Swiss law. Corn snakes were bred at the LANE, Geneva. For gene mapping analyses, we established seven families, each of which consisted of a cross between an amelanistic (*amel*/*amel* genotype) and a wild-type (WT) individual with heterozygous (*amel/*+) genotype. These seven crosses generated a total of 140 WT and 116 amelanistic F1 offspring.

### Transcriptome sequencing and assembly

RNA was extracted from multiple WT embryos (whole embryo of 10 days post oviposition, dpo; skin and muscles from embryos of 30 and 47 dpo), as well as from brain and testes of an adult WT male, using the RNeasy Plus Mini kit (Qiagen) with polyA selection. RNA quality and concentration were analysed with a NanoDrop 2000 spectrophotometer (ThermoScientific) and Qubit 2.0 Fluorometer (Life Technologies). Double-stranded cDNA was prepared with the Mint-2 cDNA synthesis kit (Evrogen) separately for the embryonic and adult tissues. Embryonic and adult cDNA were each normalised with the Trimmer-2 cDNA normalisation kit (Evrogen) and used for the preparation of two libraries with insert size of 200 bp. These were then sequenced on two lanes of an Illumina HiSeq2000 sequencer using 101 cycles per run, yielding 2.7–3.2 Gbp of high-quality (Q ≥ 30) data. The corresponding ~275 million paired-end reads were fed into the Trinity software (http://trinityrnaseq.sourceforge.net), with default parameters, to assemble the corn snake transcriptome. Trinity yielded 146,030 contigs with a cumulative length of 71.6 Mbp (N50 = 615 bp). To remove redundancy, we then used BLASTn (e-value ≤ 10^−10^) to align all contigs against each other; when a contig had multiple hits, only the largest hit of each group was included in the non-redundant set. The final non-redundant transcriptome contains 92,308 contigs with a combined length of 35.7 Mbp (N50 = 423 bp).

### Genomic DNA next-generation sequencing

Genomic DNA was extracted from blood samples of the parents and scale clips from all 256 hatchlings using QIAGEN DNeasy Blood and Tissue kits following the manufacturer’s instructions. RNA and DNA qualities and concentrations were analysed on a NanoDrop 2000 spectrophotometer (ThermoScientific) and a Qubit 2.0 Fluorometer (Life Technologies). The DNA samples from 20 WT (*amel*/+ genotype) individuals from a single family were combined in equimolar concentrations. Similarly, a second pool was made by mixing the DNA samples from 20 amelanistic (*amel*/*amel* genotype) individuals from the same single family. These two pools, as well as the individual DNA of the two parents were used to generate (using the TruSeq PE Cluster Kit v3-cBot-HS) the corresponding four genomic DNA libraries (300–400 bp insert size) that were sequenced using paired-ends 100-bp reads technology on an Illumina HiSeq2000. We sequenced the samples on three lanes, one lane for each of the two offspring pools and one lane for the indexed parental DNA samples, and obtained 20.8–34.9 Gbp of high-quality (*i.e.*, Q ≥ 30) data per lane, corresponding to an average sequencing depth of 9.5–19.4x for a haploid genome size of 1.8–2.2 Gbp (ref. 15 and Animal Genome Size Database; http://www.genomesize.com/; for closely related species of the *Elaphe* genus).

### Bioinformatic analyses: SNP calling and genomic interval mapping

To maximise the number of detected SNPs, we used the genomic reads of one parent to extend exons of the cDNA contigs with intronic and intergenic sequences in a process called exome assembly^63^. To remove assembly artifacts and chimeric sequences, we use UCLUST^72^ to cluster all sequences with at least 95% sequence identity (and kept the longest sequence). We then realigned all of the exome contigs against the non-redundant transcriptome using BLASTn (e value, ≤ 10^−5^), resulting in a final number of 138,849 exome contigs.

We then used this reference exome assembly for SNP calling. First, we mapped the genomic reads from each of the four samples (amelanistic offspring pool, WT offspring pool, WT mother, and amelanistic father) to the exome using BWA v0.7.1 (ref. 73; http://bio-bwa.sourceforge.net/bwa.shtml) with default parameters. Then, we used FreeBayes v0.9.9.2 (ref. 74; https://github.com/ekg/freebayes) with default settings to call variants. We then filtered the 510,724 reported variants by excluding (in each of the four samples) indels, triallelic SNPs, SNPs with mapping quality <100, and positions with read coverage <18 or >50. Of the resulting 197,907 SNPs, we additionally excluded those that were either fixed (or heterozygous but with the second allele supported by only one read) in the WT parent or heterozygous in the amelanistic parent. Finally, from the resulting set of 51,982 SNPs, we removed those that were either fixed (or heterozygous but with the second allele supported by only one read) in the pool of WT offspring or heterozygous in the pool of amelanistic offspring. This analysis yielded 3,278 SNPs (in 2,186 exome contigs) perfectly segregating with the expected genotype at the amelanistic locus: heterozygous in the WT parent and offspring, and fixed in the amelanistic parent and offspring. We then checked all contigs for presence of multiple SNPs and selected the 1,025 contigs in which all SNPs co-segregate. Using tBLASTx (e-value ≤ 10^−5^), we aligned these contigs against the genome assembly of *A. carolinensis* (AnoCar2.0; ref. 49) to identify the lizard genome interval orthologous to the corn snake interval containing the *amel* locus. Among the 368 contigs (=35.9%) with a hit, 146 matched to the chromosome 3 of *A. carolinensis*, of which 103 cluster in a region between 82.7 and 119.5 Mbp.

### Additional genotyping

To confirm the genomic interval identified by the procedure described above, we genotyped SNPs in selected genes of additional offspring individuals. First, all 256 offspring were genotyped for SNPs in two genes (*RAP2A* and *ARHGAP6*) flanking that interval. Then, five additional markers were genotyped in the individuals exhibiting recombination between the *amel* locus and either *RAP2A* or *ARHGAP6*. Primers used for genotyping are given in Supplementary Table S2. PCRs for SNP genotyping were carried out in 15 μl volume reactions and consisted of an initial denaturation step of 5 min at 95 °C, followed by 35 cycles of 25 s at 94 °C, 25 s at 58 °C, 60 s at 72 °C, and a final extension of 5 min at 72 °C. PCR products were purified with ExoSAP-IT (Affymetrix) and sequenced, with the primers used for PCR amplification, using the BigDye v3.1 kit on an ABI sequencer.

### Sequencing of the *OCA2* gene

To amplify the coding region of *OCA2*, we extracted total RNA from the skin of four homozygous WT (+/+) and four amelanistic (*amel*/*amel*) snakes using TriReagent Solution (Sigma Aldrich) and we synthesised cDNA with SuperScript III First-Strand System and oligo(dT)_20_ primers (Life Technologies). Overlapping fragments of *OCA2*, spanning the complete coding sequence of the *OCA2* gene, were PCR amplified from these cDNA samples and sequenced. We amplified (with the Expand Long Template PCR system; Roche Applied Science) the intron in between exons 11 and 12 of the *OCA2* gene (primers shown in Supplementary Table S2) from genomic DNA samples of homozygous WT (+/+), amelanistic (*amel*/*amel*) and heterozygous (*amel*/+) corn snakes. PCR products were visualised on 2% agarose gels and sequenced by primer walking.

## Additional Information

**How to cite this article**: Saenko, S. V. *et al.* Amelanism in the corn snake is associated with the insertion of an LTR-retrotransposon in the *OCA2* gene. *Sci. Rep.* **5**, 17118; doi: 10.1038/srep17118 (2015).

## Supplementary Material

Supplementary Information

## Figures and Tables

**Figure 1:**
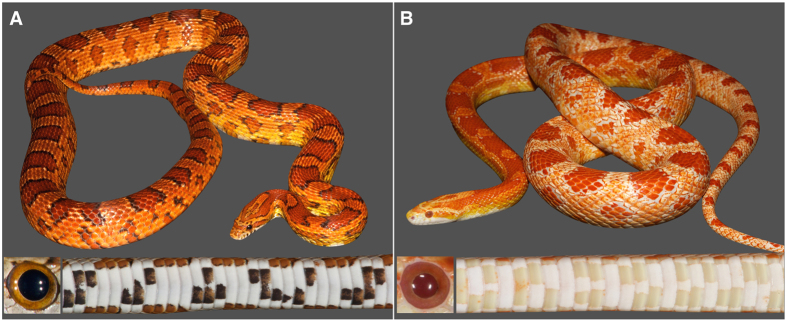
General phenotype of wild type (WT) and amelanistic corn snakes. (**A**) WT corn snakes typically exhibit, over a light orange background coloration, a pattern of dark orange dorsal saddles and lateral blotches that are outlined with black; the ventral scales are covered with a black and white checker pattern and the eyes show a black pupil and orange iris. (**B**) Amelanistic corn snakes lack all signs of melanin: the black outline of the dorsal saddles and lateral blotches is replaced by white skin, and melanin is lacking in the iris and cornea.
