# Supplementary material for: Amelanism in the corn snake is associated with the insertion of an LTR-retrotransposon in the OCA2 gene
Source: Sci Rep. 2015 Nov 24;5:17118. doi: 10.1038/srep17118 (PMC4657000; doi:10.1038/srep17118)
Supplement: Supplementary Information [file srep17118-s1.pdf]

Scientific Reports; October 08, 2015

Amelanism in the corn snake is associated with the insertion of an LTR-retrotransposon in the *OCA2* gene

SUPPLEMENTARY MATERIALS

Suzanne V. Saenko[1], Sangeet Lamichhaney[2], Alvaro Martinez Barrio[2], Nima Rafati[2], Leif Andersson[2,3,4] & Michel C. Milinkovitch[1,5,*]

[1]*Laboratory of Artificial & Natural Evolution (LANE), Department of Genetics & Evolution, University of Geneva, Switzerland.*

[2]*Science of Life Laboratory Uppsala, Department of Medical Biochemistry and Microbiology, Uppsala University, Uppsala, Sweden.*

[3]*Department of Animal Breeding and Genetics, Swedish University of Agricultural Sciences, Uppsala, Sweden.*

[4]*Department of Veterinary Integrative Biosciences, College of Veterinary Medicine and Biomedical Sciences, Texas A&M University, College Station, USA.*

[5]*SIB Swiss Institute of Bioinformatics, Geneva, Switzerland*

*Corresponding author: Michel.Milinkovitch@unige.ch ;

Tel: +41(0)22 379 67 85; Fax: +41(0)22 379 67 95

Supplementary Table S1. Absence (*'no recombination'*) or presence (*'yes'*) of recombination between the amelanistic trait and one of the seven listed genes (*RAP2A*, *UXS1*, *UNC50*, *HERC2*, *NIPA2*, *AKAP17A*, *ARHGAP6*). SNPs were genotyped by sequencing in all 256 offspring of the seven families segregating the trait.

Individual ID	Phenotype	RAP2A	UXS1	UNC50	HERC2	NIPA2	AKAP17A	ARHGAP6
266.41.01	WT	no recomb rec	no recomb	no recomb	no recomb	no recomb	no recomb	no recomb
266.41.02	WT	no recomb	no recomb	no recomb	no recomb	no recomb	no recomb	no recomb
266.41.03	WT	no recomb	no recomb	no recomb	no recomb	no recomb	no recomb	no recomb
266.41.04	WT	no recomb	no recomb	no recomb	no recomb	no recomb	no recomb	no recomb
266.41.05	WT	no recomb	no recomb	no recomb	no recomb	no recomb	no recomb	no recomb
266.41.06	WT	no recomb	no recomb	no recomb	no recomb	no recomb	no recomb	no recomb
266.41.07	WT	yes	no recomb	no recomb	no recomb	no recomb	no recomb	no recomb
266.41.08	WT	no recomb	no recomb	no recomb	no recomb	no recomb	no recomb	no recomb
266.41.09	WT	no recomb	no recomb	no recomb	no recomb	no recomb	no recomb	no recomb
266.41.10	WT	no recomb	no recomb	no recomb	no recomb	no recomb	no recomb	no recomb
266.41.11	WT	no recomb	no recomb	no recomb	no recomb	no recomb	no recomb	no recomb
266.41.12	WT	no recomb	no recomb	no recomb	no recomb	no recomb	no recomb	no recomb
266.41.13	WT	no recomb	no recomb	no recomb	no recomb	no recomb	no recomb	no recomb
266.41.14	amel	no recomb	no recomb	no recomb	no recomb	no recomb	no recomb	no recomb
266.41.15	amel	no recomb	no recomb	no recomb	no recomb	no recomb	no recomb	no recomb
266.41.16	amel	no recomb	no recomb	no recomb	no recomb	no recomb	no recomb	no recomb
266.41.17	amel	no recomb	no recomb	no recomb	no recomb	no recomb	no recomb	no recomb
266.41.18	amel	no recomb	no recomb	no recomb	no recomb	no recomb	no recomb	no recomb
266.41.19	amel	no recomb	no recomb	no recomb	no recomb	no recomb	no recomb	no recomb
266.41.20	amel	no recomb	no recomb	no recomb	no recomb	no recomb	no recomb	no recomb
266.41.21	WT	no recomb	no recomb	no recomb	no recomb	no recomb	no recomb	no recomb
266.41.22	WT	no recomb	no recomb	no recomb	no recomb	no recomb	no recomb	no recomb
266.41.23	WT	no recomb	no recomb	no recomb	no recomb	no recomb	no recomb	no recomb
266.41.24	WT	no recomb	no recomb	no recomb	no recomb	no recomb	no recomb	no recomb
266.41.25	amel	no recomb	no recomb	no recomb	no recomb	no recomb	no recomb	no recomb
266.41.26	amel	no recomb	no recomb	no recomb	no recomb	no recomb	no recomb	no recomb
266.41.27	amel	no recomb	no recomb	no recomb	no recomb	no recomb	no recomb	no recomb
266.41.28	amel	no recomb	no recomb	no recomb	no recomb	no recomb	no recomb	no recomb
266.41.29	amel	no recomb	no recomb	no recomb	no recomb	no recomb	no recomb	no recomb
266.54.01	WT	no recomb	no recomb	no recomb	no recomb	no recomb	no recomb	no recomb
266.54.02	WT	yes	no recomb	no recomb	no recomb	no recomb	no recomb	no recomb
266.54.03	WT	no recomb	no recomb	no recomb	no recomb	no recomb	no recomb	no recomb
266.54.04	WT	no recomb	no recomb	no recomb	no recomb	no recomb	no recomb	no recomb
266.54.05	WT	no recomb	no recomb	no recomb	no recomb	no recomb	no recomb	no recomb
266.54.06	WT	yes	no recomb	no recomb	no recomb	no recomb	no recomb	no recomb
266.54.07	WT	no recomb	no recomb	no recomb	no recomb	no recomb	no recomb	no recomb
266.54.08	WT	yes	yes	no recomb	no recomb	no recomb	no recomb	no recomb
266.54.09	WT	no recomb	no recomb	no recomb	no recomb	no recomb	no recomb	no recomb
266.54.10	WT	no recomb	no recomb	no recomb	no recomb	no recomb	no recomb	no recomb
266.54.11	WT	no recomb	no recomb	no recomb	no recomb	no recomb	no recomb	no recomb
266.54.12	WT	no recomb	no recomb	no recomb	no recomb	no recomb	no recomb	yes
266.54.13	amel	no recomb	no recomb	no recomb	no recomb	no recomb	no recomb	no recomb
266.54.14	amel	no recomb	no recomb	no recomb	no recomb	no recomb	no recomb	no recomb
266.54.15	amel	no recomb	no recomb	no recomb	no recomb	no recomb	no recomb	no recomb

266.54.16	amel	no recomb	no recomb	no recomb	no recomb	no recomb	no recomb	no recomb
266.54.17	amel	no recomb	no recomb	no recomb	no recomb	no recomb	no recomb	no recomb
266.54.18	amel	no recomb	no recomb	no recomb	no recomb	no recomb	no recomb	no recomb
266.54.19	amel	no recomb	no recomb	no recomb	no recomb	no recomb	no recomb	no recomb
266.54.20	amel	no recomb	no recomb	no recomb	no recomb	no recomb	no recomb	no recomb
266.54.21	amel	no recomb	no recomb	no recomb	no recomb	no recomb	no recomb	no recomb
266.54.22	amel	no recomb	no recomb	no recomb	no recomb	no recomb	no recomb	no recomb
266.54.23	amel	no recomb	no recomb	no recomb	no recomb	no recomb	no recomb	no recomb
266.54.24	amel	no recomb	no recomb	no recomb	no recomb	no recomb	no recomb	no recomb
266.54.25	amel	no recomb	no recomb	no recomb	no recomb	no recomb	no recomb	no recomb
266.54.26	amel	no recomb	no recomb	no recomb	no recomb	no recomb	no recomb	no recomb
266.54.27	WT	no recomb	no recomb	no recomb	no recomb	no recomb	no recomb	no recomb
266.54.28	WT	no recomb	no recomb	no recomb	no recomb	no recomb	no recomb	no recomb
266.54.29	WT	no recomb	no recomb	no recomb	no recomb	no recomb	no recomb	no recomb
266.54.30	WT	no recomb	no recomb	no recomb	no recomb	no recomb	no recomb	no recomb
266.54.31	WT	no recomb	no recomb	no recomb	no recomb	no recomb	no recomb	no recomb
266.54.32	WT	no recomb	no recomb	no recomb	no recomb	no recomb	no recomb	no recomb
266.54.33	WT	no recomb	no recomb	no recomb	no recomb	no recomb	no recomb	no recomb
266.54.34	WT	no recomb	no recomb	no recomb	no recomb	no recomb	no recomb	no recomb
266.54.35	WT	no recomb	no recomb	no recomb	no recomb	no recomb	no recomb	no recomb
266.54.36	WT	no recomb	no recomb	no recomb	no recomb	no recomb	no recomb	no recomb
266.54.37	WT	no recomb	no recomb	no recomb	no recomb	no recomb	no recomb	no recomb
266.54.38	amel	no recomb	no recomb	no recomb	no recomb	no recomb	no recomb	no recomb
266.54.39	amel	no recomb	no recomb	no recomb	no recomb	no recomb	no recomb	no recomb
266.54.40	amel	no recomb	no recomb	no recomb	no recomb	no recomb	no recomb	no recomb
266.54.41	amel	no recomb	no recomb	no recomb	no recomb	no recomb	yes	yes
266.54.42	amel	no recomb	no recomb	no recomb	no recomb	no recomb	no recomb	no recomb
266.54.43	amel	no recomb	no recomb	no recomb	no recomb	no recomb	no recomb	no recomb
266.54.44	amel	no recomb	no recomb	no recomb	no recomb	no recomb	no recomb	no recomb
216.56.01	WT	no recomb	no recomb	no recomb	no recomb	no recomb	no recomb	no recomb
216.56.02	WT	no recomb	no recomb	no recomb	no recomb	no recomb	no recomb	no recomb
216.56.03	WT	no recomb	no recomb	no recomb	no recomb	no recomb	no recomb	no recomb
216.56.04	WT	no recomb	no recomb	no recomb	no recomb	no recomb	no recomb	no recomb
216.56.05	WT	no recomb	no recomb	no recomb	no recomb	no recomb	no recomb	no recomb
216.56.06	WT	no recomb	no recomb	no recomb	no recomb	no recomb	no recomb	no recomb
216.56.07	amel	no recomb	no recomb	no recomb	no recomb	no recomb	no recomb	no recomb
216.56.08	amel	no recomb	no recomb	no recomb	no recomb	no recomb	no recomb	no recomb
216.56.09	amel	no recomb	no recomb	no recomb	no recomb	no recomb	no recomb	no recomb
216.56.10	amel	no recomb	no recomb	no recomb	no recomb	no recomb	no recomb	no recomb
216.56.11	amel	no recomb	no recomb	no recomb	no recomb	no recomb	no recomb	no recomb
216.56.12	amel	no recomb	no recomb	no recomb	no recomb	no recomb	no recomb	no recomb
216.56.13	amel	no recomb	no recomb	no recomb	no recomb	no recomb	no recomb	no recomb
216.56.14	amel	no recomb	no recomb	no recomb	no recomb	no recomb	no recomb	no recomb
216.56.15	amel	no recomb	no recomb	no recomb	no recomb	no recomb	no recomb	no recomb
216.56.16	WT	no recomb	no recomb	no recomb	no recomb	no recomb	no recomb	no recomb
216.56.17	WT	no recomb	no recomb	no recomb	no recomb	no recomb	no recomb	no recomb
216.56.18	WT	no recomb	no recomb	no recomb	no recomb	no recomb	no recomb	no recomb
216.56.19	amel	no recomb	no recomb	no recomb	no recomb	no recomb	no recomb	no recomb
216.56.20	amel	no recomb	no recomb	no recomb	no recomb	no recomb	no recomb	no recomb
216.56.21	amel	no recomb	no recomb	no recomb	no recomb	no recomb	no recomb	no recomb

216.56.22	amel	no recomb	no recomb	no recomb	no recomb	no recomb	no recomb	no recomb
216.56.23	amel	no recomb	no recomb	no recomb	no recomb	no recomb	no recomb	no recomb
216.56.24	WT	no recomb	no recomb	no recomb	no recomb	no recomb	no recomb	no recomb
216.56.25	WT	no recomb	no recomb	no recomb	no recomb	no recomb	no recomb	no recomb
216.56.26	WT	no recomb	no recomb	no recomb	no recomb	no recomb	no recomb	no recomb
216.56.27	WT	no recomb	no recomb	no recomb	no recomb	no recomb	no recomb	no recomb
216.56.28	WT	no recomb	no recomb	no recomb	no recomb	no recomb	no recomb	no recomb
216.56.29	WT	no recomb	no recomb	no recomb	no recomb	no recomb	no recomb	no recomb
217.57.02	WT	no recomb	no recomb	no recomb	no recomb	no recomb	no recomb	no recomb
217.57.03	WT	no recomb	no recomb	no recomb	no recomb	no recomb	no recomb	no recomb
217.57.04	WT	no recomb	no recomb	no recomb	no recomb	no recomb	no recomb	no recomb
217.57.05	WT	no recomb	no recomb	no recomb	no recomb	no recomb	no recomb	no recomb
217.57.06	WT	no recomb	no recomb	no recomb	no recomb	no recomb	no recomb	no recomb
217.57.07	WT	no recomb	no recomb	no recomb	no recomb	no recomb	no recomb	no recomb
217.57.08	WT	no recomb	no recomb	no recomb	no recomb	no recomb	no recomb	no recomb
217.57.09	WT	no recomb	no recomb	no recomb	no recomb	no recomb	no recomb	no recomb
217.57.10	WT	no recomb	no recomb	no recomb	no recomb	no recomb	no recomb	no recomb
217.57.12	WT	no recomb	no recomb	no recomb	no recomb	no recomb	no recomb	no recomb
217.57.13	WT	no recomb	no recomb	no recomb	no recomb	no recomb	no recomb	no recomb
217.57.17	amel	no recomb	no recomb	no recomb	no recomb	no recomb	no recomb	no recomb
217.57.18	amel	no recomb	no recomb	no recomb	no recomb	no recomb	no recomb	no recomb
217.57.19	amel	no recomb	no recomb	no recomb	no recomb	no recomb	no recomb	no recomb
217.57.20	amel	no recomb	no recomb	no recomb	no recomb	no recomb	no recomb	no recomb
217.57.21	amel	no recomb	no recomb	no recomb	no recomb	no recomb	no recomb	no recomb
217.57.22	amel	yes	yes	no recomb	no recomb	no recomb	no recomb	no recomb
219.58.01	amel	no recomb	no recomb	no recomb	no recomb	no recomb	no recomb	no recomb
219.58.02	WT	no recomb	no recomb	no recomb	no recomb	no recomb	no recomb	no recomb
219.58.03	WT	no recomb	no recomb	no recomb	no recomb	no recomb	no recomb	no recomb
219.58.04	amel	no recomb	no recomb	no recomb	no recomb	no recomb	no recomb	no recomb
219.58.05	amel	no recomb	no recomb	no recomb	no recomb	no recomb	no recomb	no recomb
219.58.06	WT	no recomb	no recomb	no recomb	no recomb	no recomb	no recomb	no recomb
219.58.07	amel	no recomb	no recomb	no recomb	no recomb	no recomb	no recomb	no recomb
219.58.08	amel	no recomb	no recomb	no recomb	no recomb	no recomb	no recomb	no recomb
219.58.09	amel	no recomb	no recomb	no recomb	no recomb	no recomb	no recomb	no recomb
219.58.10	amel	no recomb	no recomb	no recomb	no recomb	no recomb	no recomb	no recomb
219.58.11	amel	no recomb	no recomb	no recomb	no recomb	no recomb	no recomb	no recomb
219.58.12	amel	no recomb	no recomb	no recomb	no recomb	no recomb	no recomb	no recomb
219.58.13	amel	no recomb	no recomb	no recomb	no recomb	no recomb	no recomb	no recomb
219.58.14	amel	no recomb	no recomb	no recomb	no recomb	no recomb	no recomb	no recomb
219.58.15	WT	no recomb	no recomb	no recomb	no recomb	no recomb	no recomb	no recomb
219.58.16	WT	no recomb	no recomb	no recomb	no recomb	no recomb	no recomb	no recomb
219.58.17	WT	no recomb	no recomb	no recomb	no recomb	no recomb	no recomb	no recomb
219.58.18	WT	no recomb	no recomb	no recomb	no recomb	no recomb	no recomb	no recomb
219.58.19	WT	no recomb	no recomb	no recomb	no recomb	no recomb	no recomb	no recomb
219.58.20	WT	no recomb	no recomb	no recomb	no recomb	no recomb	no recomb	no recomb
266.41.30	WT	no recomb	no recomb	no recomb	no recomb	no recomb	no recomb	no recomb
266.41.31	amel	no recomb	no recomb	no recomb	no recomb	no recomb	no recomb	no recomb
266.41.32	amel	no recomb	no recomb	no recomb	no recomb	no recomb	no recomb	no recomb
266.41.33	WT	no recomb	no recomb	no recomb	no recomb	no recomb	no recomb	no recomb
266.41.34	WT	no recomb	no recomb	no recomb	no recomb	no recomb	no recomb	no recomb

266.41.35	amel	no recomb	no recomb	no recomb	no recomb	no recomb	no recomb	no recomb
266.41.36	amel	no recomb	no recomb	no recomb	no recomb	no recomb	no recomb	no recomb
266.41.37	amel	no recomb	no recomb	no recomb	no recomb	no recomb	no recomb	no recomb
266.41.38	WT	no recomb	no recomb	no recomb	no recomb	no recomb	no recomb	no recomb
266.41.39	amel	no recomb	no recomb	no recomb	no recomb	no recomb	no recomb	no recomb
266.41.40	WT	no recomb	no recomb	no recomb	no recomb	no recomb	no recomb	no recomb
266.41.41	WT	no recomb	no recomb	no recomb	no recomb	no recomb	no recomb	no recomb
266.41.42	amel	no recomb	no recomb	no recomb	no recomb	no recomb	no recomb	no recomb
266.41.43	WT	no recomb	no recomb	no recomb	no recomb	no recomb	no recomb	no recomb
266.41.44	amel	no recomb	no recomb	no recomb	no recomb	no recomb	no recomb	no recomb
266.41.45	WT	no recomb	no recomb	no recomb	no recomb	no recomb	no recomb	no recomb
266.41.46	WT	no recomb	no recomb	no recomb	no recomb	no recomb	no recomb	no recomb
266.41.47	WT	no recomb	no recomb	no recomb	no recomb	no recomb	no recomb	no recomb
266.41.48	WT	no recomb	no recomb	no recomb	no recomb	no recomb	no recomb	no recomb
266.54.45	WT	no recomb	no recomb	no recomb	no recomb	no recomb	no recomb	no recomb
266.54.46	WT	no recomb	no recomb	no recomb	no recomb	no recomb	no recomb	no recomb
266.54.47	WT	yes	yes	no recomb	no recomb	no recomb	no recomb	no recomb
266.54.48	WT	no recomb	no recomb	no recomb	no recomb	no recomb	no recomb	no recomb
266.54.49	WT	no recomb	no recomb	no recomb	no recomb	no recomb	no recomb	no recomb
266.54.50	WT	no recomb	no recomb	no recomb	no recomb	no recomb	no recomb	no recomb
266.54.51	WT	no recomb	no recomb	no recomb	no recomb	no recomb	no recomb	no recomb
266.54.52	WT	no recomb	no recomb	no recomb	no recomb	no recomb	no recomb	no recomb
266.54.53	WT	no recomb	no recomb	no recomb	no recomb	no recomb	no recomb	no recomb
266.54.54	amel	no recomb	no recomb	no recomb	no recomb	no recomb	no recomb	no recomb
266.54.55	amel	no recomb	no recomb	no recomb	no recomb	no recomb	no recomb	no recomb
266.54.56	amel	no recomb	no recomb	no recomb	no recomb	no recomb	no recomb	no recomb
266.54.57	amel	no recomb	no recomb	no recomb	no recomb	no recomb	no recomb	no recomb
266.54.58	amel	no recomb	no recomb	no recomb	no recomb	no recomb	no recomb	no recomb
266.54.59	amel	no recomb	no recomb	no recomb	no recomb	no recomb	no recomb	no recomb
266.54.60	amel	no recomb	no recomb	no recomb	no recomb	no recomb	no recomb	no recomb
266.54.61	amel	no recomb	no recomb	no recomb	no recomb	no recomb	no recomb	no recomb
266.54.62	amel	no recomb	no recomb	no recomb	no recomb	no recomb	no recomb	no recomb
266.54.63	amel	no recomb	no recomb	no recomb	no recomb	no recomb	no recomb	no recomb
266.54.64	amel	no recomb	no recomb	no recomb	no recomb	no recomb	no recomb	no recomb
266.54.65	amel	no recomb	no recomb	no recomb	no recomb	no recomb	no recomb	no recomb
266.62.1	WT	no recomb	no recomb	no recomb	no recomb	no recomb	no recomb	no recomb
266.62.2	WT	no recomb	no recomb	no recomb	no recomb	no recomb	no recomb	no recomb
266.62.3	amel	no recomb	no recomb	no recomb	no recomb	no recomb	no recomb	no recomb
266.62.4	amel	no recomb	no recomb	no recomb	no recomb	no recomb	no recomb	no recomb
266.62.5	amel	no recomb	no recomb	no recomb	no recomb	no recomb	no recomb	no recomb
266.62.6	WT	no recomb	no recomb	no recomb	no recomb	no recomb	no recomb	no recomb
266.62.7	WT	no recomb	no recomb	no recomb	no recomb	no recomb	no recomb	no recomb
266.62.8	WT	no recomb	no recomb	no recomb	no recomb	no recomb	no recomb	no recomb
266.62.9	WT	no recomb	no recomb	no recomb	no recomb	no recomb	no recomb	no recomb
266.62.10	WT	no recomb	no recomb	no recomb	no recomb	no recomb	no recomb	no recomb
266.62.11	amel	no recomb	no recomb	no recomb	no recomb	no recomb	no recomb	no recomb
266.62.12	WT	no recomb	no recomb	no recomb	no recomb	no recomb	no recomb	no recomb
266.62.13	WT	no recomb	no recomb	no recomb	no recomb	no recomb	no recomb	no recomb
266.62.14	WT	no recomb	no recomb	no recomb	no recomb	no recomb	no recomb	no recomb
266.62.15	WT	no recomb	no recomb	no recomb	no recomb	no recomb	no recomb	no recomb

266.62.16	amel	no recomb	no recomb	no recomb	no recomb	no recomb	no recomb	no recomb
266.62.17	amel	yes	yes	no recomb	no recomb	no recomb	no recomb	no recomb
266.62.18	amel	no recomb	no recomb	no recomb	no recomb	no recomb	no recomb	no recomb
266.62.19	WT	no recomb	no recomb	no recomb	no recomb	no recomb	no recomb	no recomb
61.267.1	WT	no recomb	no recomb	no recomb	no recomb	no recomb	no recomb	no recomb
61.267.2	WT	no recomb	no recomb	no recomb	no recomb	no recomb	no recomb	no recomb
61.267.3	WT	no recomb	no recomb	no recomb	no recomb	no recomb	no recomb	no recomb
61.267.4	WT	no recomb	no recomb	no recomb	no recomb	no recomb	no recomb	no recomb
61.267.5	WT	no recomb	no recomb	no recomb	no recomb	no recomb	no recomb	no recomb
61.267.6	WT	no recomb	no recomb	no recomb	no recomb	no recomb	no recomb	no recomb
61.267.7	WT	no recomb	no recomb	no recomb	no recomb	no recomb	no recomb	no recomb
61.267.8	WT	no recomb	no recomb	no recomb	no recomb	no recomb	no recomb	no recomb
61.267.9	WT	no recomb	no recomb	no recomb	no recomb	no recomb	no recomb	no recomb
61.267.10	amel	no recomb	no recomb	no recomb	no recomb	no recomb	no recomb	no recomb
61.267.12	amel	no recomb	no recomb	no recomb	no recomb	no recomb	no recomb	no recomb
61.267.13	amel	no recomb	no recomb	no recomb	no recomb	no recomb	no recomb	no recomb
61.267.14	amel	no recomb	no recomb	no recomb	no recomb	no recomb	no recomb	no recomb
61.267.15	amel	no recomb	no recomb	no recomb	no recomb	no recomb	no recomb	no recomb
61.267.16	amel	no recomb	no recomb	no recomb	no recomb	no recomb	no recomb	no recomb
216.56.30	WT	no recomb	no recomb	no recomb	no recomb	no recomb	no recomb	no recomb
216.56.31	WT	no recomb	no recomb	no recomb	no recomb	no recomb	no recomb	no recomb
216.56.32	WT	no recomb	no recomb	no recomb	no recomb	no recomb	no recomb	no recomb
216.56.33	WT	no recomb	no recomb	no recomb	no recomb	no recomb	no recomb	no recomb
216.56.34	WT	no recomb	no recomb	no recomb	no recomb	no recomb	no recomb	no recomb
216.56.35	WT	no recomb	no recomb	no recomb	no recomb	no recomb	no recomb	no recomb
216.56.36	WT	no recomb	no recomb	no recomb	no recomb	no recomb	no recomb	no recomb
216.56.37	WT	no recomb	no recomb	no recomb	no recomb	no recomb	no recomb	no recomb
216.56.38	WT	no recomb	no recomb	no recomb	no recomb	no recomb	no recomb	no recomb
216.56.39	amel	no recomb	no recomb	no recomb	no recomb	no recomb	no recomb	no recomb
216.56.40	amel	no recomb	no recomb	no recomb	no recomb	no recomb	no recomb	no recomb
216.56.41	amel	no recomb	no recomb	no recomb	no recomb	no recomb	no recomb	no recomb
216.56.42	amel	no recomb	no recomb	no recomb	no recomb	no recomb	no recomb	no recomb
216.56.43	amel	no recomb	no recomb	no recomb	no recomb	no recomb	no recomb	no recomb
216.56.44	amel	no recomb	no recomb	no recomb	no recomb	no recomb	no recomb	no recomb
216.56.45	amel	no recomb	no recomb	no recomb	no recomb	no recomb	no recomb	no recomb
216.56.46	amel	no recomb	no recomb	no recomb	no recomb	no recomb	no recomb	no recomb
217.57.26	WT	no recomb	no recomb	no recomb	no recomb	no recomb	no recomb	no recomb
217.57.27	amel	no recomb	no recomb	no recomb	no recomb	no recomb	no recomb	no recomb
217.57.28	WT	no recomb	no recomb	no recomb	no recomb	no recomb	no recomb	no recomb
217.57.29	amel	no recomb	no recomb	no recomb	no recomb	no recomb	no recomb	no recomb
217.57.30	WT	no recomb	no recomb	no recomb	no recomb	no recomb	no recomb	no recomb
217.57.31	amel	no recomb	no recomb	no recomb	no recomb	no recomb	no recomb	no recomb
217.57.32	WT	no recomb	no recomb	no recomb	no recomb	no recomb	no recomb	no recomb
217.57.33	amel	no recomb	no recomb	no recomb	no recomb	no recomb	no recomb	no recomb
217.57.34	amel	no recomb	no recomb	no recomb	no recomb	no recomb	no recomb	no recomb
217.57.35	WT	no recomb	no recomb	no recomb	no recomb	no recomb	no recomb	no recomb
217.57.36	amel	no recomb	no recomb	no recomb	no recomb	no recomb	no recomb	no recomb
217.57.37	WT	no recomb	no recomb	no recomb	no recomb	no recomb	no recomb	no recomb
217.57.38	WT	no recomb	no recomb	no recomb	no recomb	no recomb	no recomb	no recomb
217.57.39	WT	no recomb	no recomb	no recomb	no recomb	no recomb	no recomb	no recomb

217.57.40	WT	no recomb	no recomb	no recomb	no recomb	no recomb	no recomb	no recomb
217.57.41	WT	no recomb	no recomb	no recomb	no recomb	no recomb	no recomb	no recomb
217.57.42	WT	no recomb	no recomb	no recomb	no recomb	no recomb	no recomb	no recomb
217.57.43	amel	no recomb	no recomb	no recomb	no recomb	no recomb	no recomb	no recomb
217.57.44	amel	no recomb	no recomb	no recomb	no recomb	no recomb	no recomb	no recomb
217.57.45	WT	no recomb	no recomb	no recomb	no recomb	no recomb	no recomb	no recomb
217.57.46	amel	no recomb	no recomb	no recomb	no recomb	no recomb	no recomb	no recomb
217.57.47	WT	no recomb	no recomb	no recomb	no recomb	no recomb	no recomb	no recomb
217.57.48	amel	no recomb	no recomb	no recomb	no recomb	no recomb	no recomb	no recomb
217.57.49	amel	no recomb	no recomb	no recomb	no recomb	no recomb	no recomb	no recomb
217.57.50	WT	no recomb	no recomb	no recomb	no recomb	no recomb	no recomb	no recomb
217.57.51	WT	no recomb	no recomb	no recomb	no recomb	no recomb	no recomb	no recomb

Supplementary Table S2. List of primers used for genotyping SNPs (in seven genes in the interval comprising the amelanistic locus) and for amplifying (long-range PCR) from genomic DNA the 11th intron of the *OCA2* gene.

Primer	Sequence 5'-3'	Purpose	Gene
rap2a.F	AGGGCTGGTTTGGTATCTTGA	genotyping	rap2a
rap2a.R	CTGTGCTGAAGACCACCCTA	genotyping	rap2a
uxs1.F	ACTGCTTGGGACTGAGGATC	genotyping	uxs1
uxs1.R	ACATGAAGAGCTGGGTATACTG	genotyping	uxs1
herc2.F	AGCAACACAGACCATCCACT	genotyping	herc2
herc2.R	AGGCCAACACTCATTTCCATAT	genotyping	herc2
nipa2.F	TCGAAGAGCAGTGGTGGATT	genotyping	nipa2
nipa2.R	TGCAAACACACCTAAGCCTG	genotyping	nipa2
unc50.F	CTGCCATTTCCTTCAGTGGT	genotyping	unc50
unc50.R	TGAGGCCAACAGATTCACATG	genotyping	unc50
akap17a.F	TAAAGCAGCCAGGGAAATCC	genotyping	akap17a
akap17a.R	CATCCCTCGCAAAGCATTCA	genotyping	akap17a
arhgap6.F	GGTTATGGCAGATGTTCACCC	genotyping	arhgap6
arhgap6.R	ACTTACTCTTCTCCGTGCCC	genotyping	arhgap6
oca2_exon11.F	AGTGGCATGGATTGATTTCGA	long-range PCR	oca2
oca2_exon12.R	GTTTCAGAGAAGATAGCAACC	long-range PCR	oca2

Supplementary Data S1. Sequence of the wild-type (WT) and amel alleles of the corn snake OCA2 cDNA.

```
>Oca2-cDNA-WT
AACACAATTCATCTGGTACCAGAACTATGTACCTGGACAACAAAGATGATGGTGCAATTA
CCAGAAACATAGATATGGAATTGGACTCTCACCACATGGATGTGCTTGGAGATGGAGCAA
CAGAGCCAATTTCTTACAATGTCAGCAMACTGAAACTCTTGAAGGAGATCATGATTGGTC
AGGGARCATCACATCAACCCTATCCAAGAATTGCACAAGAGCCAGACCAGTGTATTACTT
TGGGGAAAGAATTCAGTCCTYTCACTATGCAACAGAGGTATCATTACAGTTTGCCAAAAT
TCTTCAGCACAAGATCAGAAGATAACTGTTTTACAGAAAGGACTCCTCTGATGAAAGCTT
CCACTAAAAGCAATGGGTTAAGATGTACAGATGTTCACAATACAGATTTCATCACAGATG
ATGAGTCTTGGGAAAACAGTTCTGCCGAATTTGAGCAGAGGTCTCAGCCAGGAAGTGAAA
TGACCAGCTTGTCCAGATCTGCTTCCTGCTCAGAAAAATGTGAAATATTGGACAATTTTC
ATATCAAATTCAATTTATCCAAGATGAGATGCTGCTTAAAACTCCTAAAGGTTTCATGCC
TTTTTATMTTTGTAGTTATATGCTCTATTTTGTTTAGTATTCATCCTGAAAACAGAACAT
CTTGGCAGATGCTGGCTGTTTCACCTATGGACATTTACACTGGGAACTTTAGCAGTTTCA
GTGATTCTGCTCTTTTAAAGGTAGAACTGGGAGGGCCCTTTGTAACYGAGCGACAMCCAA
AAGACTATATTGTAGTTCAAATTGGACAAATAGAAGACACCWGTCCTAAAAGGAGACGTC
AACAGCAAATCTTATATAATTGGACTCTTGCATTAAAYCTGAAGAAAAATGAGCAAGTTA
TTGTGACTAGAATCTTCGAGACAATGAATAGAAAAGAAAGCTCCCTAAGAATCCAGGCAT
TTCTTCAGGAATCAGAAATCATTCCWCTTTCTATGACACATCAGTATCTTCATGCAAATA
TAGAGGCTCAAGTCACAATAGCTTCAGTCATTTTAGCAGGTGTTTATGTTCTGATCGTGT
TTGAGATTGTCCACAGGACATTAGCTGCAATGTTGGGATCTTTGGCTGCCTTGGCTGCCT
TAGCTGCAATTGGTGATAAGCCGAGCYTGATCAAAGTAGTGGCATGGATTGATTTCGAAA
CTCTGGCACTATTGTTTGGAATG~~~~~~~~~~~~~~~~~~~~~~~~~~~~~~~~~~~~~
~~~~~~~~~~~~~~~~~~~~~~~~~~~~~~~~~~~~~~~~~~~~~~~~~~~~~~~~~~~~
~~~~~~~~~~~~~~~~~~~~~~~~~~~~~~~~~~~~~~~~~~~~~~~~~~~~~~~~~~~~
~~~~~~~~~~~~~~~~~~~~~~~~~~~~~~~~~~~~~~~~~~~~~~~~~~~~~~~~~~~~
~~~~~~~~~~~~~~~~~~~~~~~~~~~~~~~~~~~~~~~~~~~~~~~~~~~~~~~~~~~~
~~~~~~~~~~~~~~~~~~~~~~~~~~~~~~~~~~~~~~~~~~~~~~~~~~~~~~~~~~~~
~~~~~~~~~~~~~~~~~~~~~~~~~~~~~~~~~~~~~~~~~~~~~~~~~~~~~~~~~~~~
ATGCTTTTGGTTGCTATCTTCTCTGAAACTGGGTTTTTTGATTACTGTGCAGTAAAGGCT
TATAGACTTTCTCGAGGTAAAGTGTGGGCCATGATTATCATTCTCTGTTTTTTTGCTGCA
ATTCTATCAGCCTTTCTGGACAATGTTACTACGGTGCTCCTCTTTACTCCAGTAACTATA
AGRTTATGTGAGGTGCTCAATCTTGATCCCAGGCATGTTCTGATTGCTGAAGTTATCTTC
ACAAATATTGGCGGGGCTGCTACAGCTATTGGGGATCCTCCAAATGTGATTATTGTTTCA
AATCAGGAACTCAGGAAGATGGGGTTGGACTTTGCTGCATTTACGGGGCATATGTTTATT
GGAATTTGTCTTGTACTTCTGGTTTCCTTTCCTTTTCTACGGCTACTTTACTGGAATAAG
AAACTCTATAACAAAGAGCCAAGTGAGATTGTTGAACTGAAACATGAAATCCATGTTTGG
AAGCTGACAGCCCAGAGAATTAACCCTGCAAGCAGAGAAGAAACAGCTGTGAAGTGCCTC
TTAATGCAAAAAGTTAGAACTCTAGAGTTTTTGCTAAGGAAAAAGCTYAAAACTTTCCAC
AGACAAATCTCACAAGAAGATAAAAACTGGGAAACAAATATCCAGGAGCTCCAAAAAAAG
CACAGAATAACAGACAAGTTGCTTCTCATCAAATGCCTGATGGTTTTAGGAATTGTKATT
```

CTCATGTACTTTCTCAATTCATTTGTTCCAGGAATTCATTTAGATCTTGGATGGATTGCT
ATGTTGGGAGCTGTGTGGCTTCTTGTTCTAGCCAACATTCATGATTTTGAAATGATTCTG
AACAGAGTGGAATGGGCAACCCTCCTTTTCTTTGCAGCATTATTTGTTTTAATGGAGGCT
TTGGCTCATCTTCATTTAATAGAATACATAGGAGAACAAAYAGCTTTGTTGATAAAGGTA
GTCCCTGAAGAACAACGCTTGACAGTTGCTATTATTTTAATTCTGTGGGTCTCTGCATTG
GCATCATCTCWAATTGACAATATTCCATTCACTGCTACAATGATTCCTGTACTTCTCAAT
CTAAGTCAAGATCCTGAGGTTAACTTACCTGTAAAACCACTGATCTTTGCATTAGCCATA
GGTGCCTGTCTTGGAGGTAATGGGACATTGATTGGAGCCTCTGCAAATGTCGTTTGTGCA
GGCATTGCAGAACAGCATGGCTATGGCTTTTCTTTCATGGAATTTTTCAGGTTGGGATTC
CCCATGACTGTCATATCCTGTACCATTGGAATGTGTTATCTTCTTGTTGCTCATGTTGTA
TTGGGATGGGCTTAAATTTTATATGTTGATTATGTACTATATGAACAAAATATTCTGTCA
AATGTTTTCAGCTTTTGTTAAATCTTTTGAACAATGAAAGAAAACCAGATTTTTTTTTTT
AAAAAAAATGTTCCAACTTGGCTTTCCATCATGTGCAAAAAAATCTAGTTTCTGAATGAA
ATCAAGCATTGATTTTGGTAGGAAAATTTTTTTAGTCTGTGCTCCCTATGAGA
>Oca2-cDNA-amel
AACACAATTCATCTGGTACCAGAACTATGTACCTGGACAACAAAGATGATGGTGCAATTA
CCAGAAACATAGATATGGAATTGGACTCTCACCACATGGATGTGCTTGGAGATGGAGCAA
CAGAGCCAATTTCTTACAATGTCAGCAAACTGAAACTCTTGAAGGAGATCATGATTGGTC
AGGGAGCATCACATCAACCCTATCCAAGAATTGCACAAGAGCCAGACCAGTGTATTACTT
TGGGGAAAGAATTCAGTCCTCTCACTATGCAACAGAGGTATCATTACAGTTTGCCAAAAT
TCTTCAGCACAAGATCAGAAGATAACTGTTTTACAGAAAGGACTCCTCTGATGAAAGCTT
CCACTAAAAGCAATGGGTTAAGATGTACAGATGTTCACAATACAGATTTCATCACAGATG
ATGAGTCTTGGGAAAACAGTTCTGCCGAATTTGAGCAGAGGTCTCAGCCAGGAAGTGAAA
TGACCAGCTTGTCCAGATCTGCTTCCTGCTCAGAAAAATGTGAAATATTGGACAATTTTC
ATATCAAATTCAATTTATCCAAGATGAGATGCTGCTTAAAACTCCTAAAGGTTTCATGCC
TTTTTATCTTTGTAGTTATATGCTCTATTTTGTTTAGTATTCATCCTGAAAACAGAACAT
CTTGGCAGATGCTGGCTGTTTCACCTATGGACATTTACACTGGGAACTTTAGCAGTTTCA
GTGATTCTGCTCTTTTAAAGGTAGAACTGGGAGGGCCCTTTGTAACCGAGCGACAACCAA
AAGACTATATTGTAGTTCAAATTGGACAAATAGAAGACACCAGTCCTAAAAGGAGACGTC
AACAGCAAATCTTATATAATTGGACTCTTGCATTAAATCTGAAGAAAAATGAGCAAGTTA
TTGTGACTAGAATCTTCGAGACAATGAATAGAAAAGAAAGCTCCCTAAGAATCCAGGCAT
TTCTTCAGGAATCAGAAATCATTCCTCTTTCTATGACACATCAGTATCTTCATGCAAATA
TAGAGGCTCAAGTCACAATAGCTTCAGTCATTTTAGCAGGTGTTTATGTTCTGATCGTGT
TTGAGATTGTCCACAGGACATTAGCTGCAATGTTGGGATCTTTGGCTGCCTTGGCTGCCT
TAGCTGCAATTGGTGATAAGCCGAGCTTGATCAAAGTAGTGGCATGGATTGATTTCGAAA
CTCTGGCACTATTGTTTGGAATGGAACACAATTAGACAGGCACTAATCTGGGAGGAGCAA
GAACTTATATACAGAGAAGGCGGGGCTAATGGCTCGTTCTGGGCATGCTCAGTCCTCATT
TTTCTCCAGCAGTCGATGAGGGAGGTTCCTGTGTCTGCAGCTCCTGGGGTGAAGTCCCAG
ACGGCCTTCTGTTGCTCTGCTTTGTTTCCGGAGGGCCTCTGGTGACTGAAGAATGCCTGG
GGTGCTGCTCTGCTCATCGGGGAGTCTTCTCTGAGGTCGTCAGTAAGTGGAGCTTGAGGC
GGGCCGCCATTGCCAATGGAGCCGGGTTGTTCAGGGAGCTGCTCTTCAGTAGGAACACAA
TTAGACAGGCACTAATCTGGGAGGAGCAAGAACTTATATACAGAGAAGGCGGGGCTAATG
ATGCTTTTGGTTGCTATCTTCTCTGAAACTGGGTTTTTTGATTACTGTGCAGTAAAGGCT
TATAGACTTTCTCGAGGTAAAGTGTGGGCCATGATTATCATTCTCTGTTTTTTTGCTGCA

ATTCTATCAGCCTTTCTGGACAATGTTACTACGGTGCTCCTCTTTACTCCAGTAACTATA
AGGTTATGTGAGGTGCTCAATCTTGATCCCAGGCATGTTCTGATTGCTGAAGTTATCTTC
ACAAATATTGGCGGGGCTGCTACAGCTATTGGGGATCCTCCAAATGTGATTATTGTTTCA
AATCAGGAACTCAGGAAGATGGGGTTGGACTTTGCTGCATTTACGGGGCATATGTTTATT
GGAATTTGTCTTGTACTTCTGGTTTCCTTTCCTTTTCTACGGCTACTTTACTGGAATAAG
AAACTCTATAACAAAGAGCCAAGTGAGATTGTTGAACTGAAACATGAAATCCATGTTTGG
AAGCTGACAGCCCAGAGAATTAACCCTGCAAGCAGAGAAGAAACAGCTGTGAAGTGCCTC
TTAATGCAAAAAGTTAGAACTCTAGAGTTTTTGCTAAGGAAAAAGCTCAAAACTTTCCAC
AGACAAATCTCACAAGAAGATAAAAACTGGGAAACAAATATCCAGGAGCTCCAAAAAAAG
CACAGAATAACAGACAAGTTGCTTCTCATCAAATGCCTGATGGTTTTAGGAATTGTTATT
CTCATGTACTTTCTCAATTCATTTGTTCCAGGAATTCATTTAGATCTTGGATGGATTGCT
ATGTTGGGAGCTGTGTGGCTTCTTGTTCTAGCCAACATTCATGATTTTGAAATGATTCTG
AACAGAGTGGAATGGGCAACCCTCCTTTTCTTTGCAGCATTATTTGTTTTAATGGAGGCT
TTGGCTCATCTTCATTTAATAGAATACATAGGAGAACAAACAGCTTTGTTGATAAAGGTA
GTCCCTGAAGAACAACGCTTGACAGTTGCTATTATTTTAATTCTGTGGGTCTCTGCATTG
GCATCATCTCTAATTGACAATATTCCATTCACTGCTACAATGATTCCTGTACTTCTCAAT
CTAAGTCAAGATCCTGAGGTTAACTTACCTGTAAAACCACTGATCTTTGCATTAGCCATA
GGTGCCTGTCTTGGAGGTAATGGGACATTGATTGGAGCCTCTGCAAATGTCGTTTGTGCA
GGCATTGCAGAACAGCATGGCTATGGCTTTTCTTTCATGGAATTTTTCAGGTTGGGATTC
CCCATGACTGTCATATCCTGTACCATTGGAATGTGTTATCTTCTTGTTGCTCATGTTGTA
TTGGGATGGGCTTAAATTTTATATGTTGATTATGTACTATATGAACAAAATATTCTGTCA
AATGTTTTCAGCTTTTGTTAAATCTTTTGAACAATGAAAGAAAACCAGATTTTTTTTTTT
AAAAAAAATGTTCCAACTTGGCTTTCCATCATGTGCAAAAAAAATCTGGTTTCTGAATGAA
ATCAAGCATTGATTTTGGTAGGAAAATTTTTTTAGTCTGTGCTCCCTATGAGA

Supplementary Data S2. Sequence of the wild-type (WT) and amel alleles of the corn snake OCA2 11th intron.

>Oca2-intron11-WT
AGTGGCATGGATTGATTTCGAAACTCTGGCACTATTGTTTGGAATGgtaactgaatgttt
tcatgacaccttaaaagatctgaaattttaaaaaaatactatatataaactctacaggtt
actatcattcatatgtgtggttgggcagaaagagtttcagcgttagctcctttttaaact
gttttggcttaaaatgttaagtatgagtcat~~~~~~~~~~~~~~~~~~~~~~~~~~~~~
~~~~~~
~~~~~~
~~~~~~
~~~~~~
~~~~~~
~~~~~~
~~~~~~
~~~~~~
~~~~~~
~~~~~~

```
~~~~~~~~~~~~~~~~~~~~~~~~~~~~~~~~~~~~~~~~~~~~~~~~~~~~~~~~~~~~
~~~~~~~~~~~~~~~~~~~~~~~~~~~~~~~~~~~~~~~~~~~~~~~~~~~~~~~~~~~~
~~~~~~~~~~~~~~~~~~~~~~~~~~~~~~~~~~~~~~~~~~~~~~~~~~~~~~~~~~~~
~~~~~~~~~~~~~~~~~~~~~~~~~~~~~~~~~~~~~~~~~~~~~~~~~~~~~~~~~~~~
~~~~~~~~~~~~~~~~~~~~~~~~~~~~~~~~~~~~~~~~~~~~~~~~~~~~~~~~~~~~
~~~~~~~~~~~~~~~~~~~~~~~~~~~~~~~~~~~~~~~~~~~~~~~~~~~~~~~~~~~~
~~~~~~~~~~~~~~~~~~~~~~~~~~~~~~~~~~~~~~~~~~~~~~~~~~~~~~~~~~~~
~~~~~~~~~~~~~~~~~~~~~~~~~~~~~~~~~~~~~~~~~~~~~~~~~~~~~~~~~~~~
~~~~~~~~~~~~~~~~~~~~~~~~~~~~~~~~~~~~~~~~~~~~~~~~~~~~~~~~~~~~
~~~~~~~~~~~~~~~~~~~~~~~~~~~~~~~~~~~~~~~~~~~~~~~~~~~~~~~~~~~~
~~~~~~~~~~~~~~~~~~~~~~~~~~~~~~~~~~~~~~~~~~~~~~~~~~~~~~~~~~~~
~~~~~~~~~~~~~~~~~~~~~~~~~~~~~~~~~~~~~~~~~~~~~~~~~~~~~~~~~~~~
~~~~~~~~~~~~~~~~~~~~~~~~~~~~~~~~~~~~~~~~~~~~~~~~~~~~~~~~~~~~
~~~~~~~~~~~~~~~~~~~~~~~~~~~~~~~~~~~~~~~~~~~~~~~~~~~~~~~~~~~~
~~~~~~~~~~~~~~~~~~~~~~~~~~~~~~~~~~~~~~~~~~~~~~~~~~~~~~~~~~~~
~~~~~~~~~~~~~~~~~~~~~~~~~~~~~~~~~~~~~~~~~~~~~~~~~~~~~~~~~~~~
~~~~~~~~~~~~~~~~~~~~~~~~~~~~~~~~~~~~~~~~~~~~~~~~~~~~~~~~~~~~
~~~~~~~~~~~~~~~~~~~~~~~~~~~~~~~~~~~~~~~~~~~~~~~~~~~~~~~~~~~~
~~~~~~~~~~~~~~~~~~~~~~~~~~~~~~~~~~~~~~~~~~~~~~~~~~~~~~~~~~~~
~~~~~~~~~~~~~~~~~~~~~~~~~~~~~~~~~~~~~~~~~~~~~~~~~~~~~~~~~~~~
~~~~~~~~~~~~~~~~~~~~~~~~~~~~~~~~~~~~~~~~~~~~~~~~~~~~~~~~~~~~
~~~~~~~~~~~~~~~~~~~~~~~~~~~~~~~~~~~~~~~~~~~~~~~~~~~~~~~~~~~~
~~~~~~~~~~~~~~~~~~~~~~~~~~~~~~~~~~~~~~~~~~~~~~~~~~~~~~~~~~~~
~~~~~~~~~~~~~~~~~~~~~~~~~~~~~~~~~~~~~~~~~~~~~~~~~~~~~~~~~~~~
~~~~~~~~~~~~~~~~~~~~~~~~~~~~~~~~~~~~~~~~~~~~~~~~~~~~~~~~~~~~
~~~~~~~~~~~~~~~~~~~~~~~~~~~~~~~~~~~~~~~~~~~~~~~~~~~~~~~~~~~~
~~~~~~~~~~~~~~~~~~~~~~~~~~~~~~~~~~~~~~~~~~~~~~~~~~~~~~~~~~~~
~~~~~~~~~~~~~~~~~~~~~~~~~~~~~~~~~~~~~~~~~~~~~~~~~~~~~~~~~~~~
~~~~~~~~~~~~~~~~~~~~~~~~~~~~~~~~~~~~~~~~~~~~~~~~~~~~~~~~~~~~
~~~~~~~~~~~~~~~~~~~~~~~~~~~~~~~~~~~~~~~~~~~~~~~~~~~~~~~~~~~~
~~~~~~~~~~~~~~~~~~~~~~~~~~~~~~~~~~~~~~~~~~~~~~~~~~~~~~~~~~~~
~~~~~~~~~~~~~~~~~~~~~~~~~~~~~~~~~~~~~~~~~~~~~~~~~~~~~~~~~~~~
~~~~~~~~~~~~~~~~~~~~~~~~~~~~~~~~~~~~~~~~~~~~~~~~~~~~~~~~~~~~
~~~~~~~~~~~~~~~~~~~~~~~~~~~~~~~~~~~~~~~~~~~~~~~~~~~~~~~~~~~~
~~~~~~~~~~~~~~~~~~~~~~~~~~~~~~~~~~~~~~~~~~~~~~~~~~~~~~~~~~~~
~~~~~~~~~~~~~~~~~~~~~~~~~~~~~~~~~~~~~~~~~~~~~~~~~~~~~~~~~~~~
~~~~~~~~~~~~~~~~~~~~~~~~~~~~~~~~~~~~~~~~~~~~~~~~~~~~~~~~~~~~
~~~~~~~~~~~~~~~~~~~~~~~~~~~~~~~~~~~~~~~~~~~~~~~~~~~~~~~~~~~~
~~~~~~~~~~~~~~~~~~~~~~~~~~~~~~~~~~~~~~~~~~~~~~~~~~~~~~~~~~~~
~~~~~~~~~~~~~~~~~~~~~~~~~~~~~~~~~~~~~~~~~~~~~~~~~~~~~~~~~~~~
~~~~~~~~~~~~~~~~~~~~~~~~~~~~~~~~~~~~~~~~~~~~~~~~~~~~~~~~~~~~
~~~~~~~~~~~~~~~~~~~~~~~~~~~~~~~~~~~~~~~~~~~~~~~~~~~~~~~~~~~~
~~~~~~~~~~~~~~~~~~~~~~~~~~~~~~~~~~~~~~~~~~~~~~~~~~~~~~~~~~~~
~~~~~~~~~~~~~~~~~~~~~~~~~~~~~~~~~~~~~~~~~~~atcccaaatatggcagg
```

gaactggtcacaaatatgtaccactagcctgccggtccctattttgggctctccaatccc
ttcaggctttttcaggtcagcccttttccagggtatcagggtgtaattatctccctgaga
gaaaatgtcttacctggggcaagtagttaaactgttaccacttggttttaggtgaagaaa
tgctgtttcttcccaatcctttctctgaatctggatgggaaagaacaggcaattactctt
ctcagtggcttaatttctaagcacctagctttgatttccctctctaaaacgatccaagat
ctttttagtttagctgttgagcaatgccaggtccagtcagaaaatctgtttctgtttcta
cactccttcttattctgaattctgctgccaataaagatattgctaactacctctatttgt
gagagcttttacagagatagactatttctacatcatctcctagttggcaggccactccat
tcacaactagaacgttactgaatttggatcagttcaaggatggctgtttctgagctagtt
gttgaaaaagagcaatcttcattctttcactatatactgtagatgagaatgtatgctgtg
agtccaagatcttgctttacatgcttatagtttctaaatgtcttgattttggcctggtgg
tatttctcattagagttaattattatgtataaatgaataaatctgctttgaaaaatctct
ccattaactaaagtgaaaaaaaatatcaacaaatggctatactttttctgcataaaacac
agttggatatgatttagttccatagtagctccctatgggaatataaaccagggaatcagc
agcattttcaccccagagtgctagataaagtgggagtgagagatactcaaatgtacatat
gtaggaatatgtagattttgacgacgacccccctggtaagtcaacatgtggtccctgtgac
catcctggtggaattagtggtgccctcattgggcacccaggggaggatgttggccctatt
ggttttgggttgcaccaggtgtatggttaggttgtagagaaattgggactgagactgtgg
agattgaggatgcccatagccgaatgacaattggatggattggtggtgggtgggtgggtg
gatgggtgaggaagctcacttcataatggaacttgtggaaatgaggatggggagacacac
agagtttctgagttttatagtagcacctgaaacatttactaagtgagtcctccttctatg
tcctgttttcctgaaaatgttcagtagtgtaagtgcatttgggtgaggcaaagggcagaa
ggaacacataattggctacacagaagtcttgaaatattagagaaataaaagtcactggta
gagtactaccaagttgcaagaatatagcattaatcccaaagttgcagcttctgcctccca
atggctagcaattttccttttaactggcataggaatgattcaaacctgtatcaatattgg
gtttatccaaaactgaaatactttcaaaagaaagatttaggaataatctactcgctccgt
gaccattcatatgttttaagatctttcattaaagtggatgcacattcctatccactctaa
aaaaagaagcccccaaataattgttttcaactctgaaaaagaatttgcactgaaaatctg
ctctgggattcatttaatgacattagttcactgaaggaaaatggcacgttcaatttcatc
tgaaagattctccaaaagagtcatatttacctatgtaaaacaaattcaaacatagcagca
cagctcttttaaaagatttctgtctttctagATGCTTTTGGTTGCTATCTTCTCTGAAAC
TGGGTTTTTTGATTACTGTGCAGTAAAG
>Oca2-intron11-amel
AGTGGCATGGATTGATTTCGAAACTCTGGCACTATTGTTTGGAATGgtaactgaatgttt
tcatgacaccttaaaagatctgaaattttaaaaaaatactatatataaactctacaggtt
actatcattcatatgtgtggttgggcagaaagagtttcagcgttagctcctttttaaact
gttttggcttaaaatgttaagtatgagtcatatccctgttgctagtttgattatcctggt
taggcccatatataagagactgtaggctttcttgtaatagtaatatttatttcttttctt
cctcagGAACACAATTAGACAGGCACTAATCTGGGAGGAGCAAGAACTTATATACAGAGA
AGGCGGGGCTAATGgtaagtactaaccaatggtaagtgacacaatgacatcacacaatga
catataaacaagcacacatatttacagatatcaggttaggtattttcatctgtaacaaat
ccccctctaaaatacctaactaatggtgggttattaagattatatatgcacatcaagttt
ggaagtcaaatacatatgttgtttggttggcaatggcttagtgaggatatcagcaatttg
ttcactagtattgcaatggatagctttaataaatcctatacttgacagttgtcttacatg

tttatatttatatcgatatgtcgagatcgttgacctattgcttcggaagctgatatatc
aattgctgccttattgtcctcattgataataattggataaacaggttcatcccacacatt
ggttaacaagtcatcgaaccaagttagattgttgaccgctttggataaacaaacatattc
ggcttccgctgtggaagtggcaattaaattctgtttgcctgtgtaccagtgcaccgcaca
gccatttataagaaaaacatatccagaagtactttttctggtttttgaatcagtagccca
atcagcatcagcgtagcaggtcaaagttggtttttcagaaatgaatggttctaagaataa
gcacatatcaatgctagactttagatattttagaatcctcttaattccttcccagtggta
tgagttaggattgcttacatgtcttgacaacaaatttacagcaagggtgatatcaggcct
ggtccaattagatatgtataataaggacccaataattgatcgatataacgtgttatcctt
gaatggttcgtactcattatacacattcctataaaaatcagttgacataggagtatcaca
aatttttgaattaattaaattacatttttgtatgagttccataatcttgtctttctgaga
taatttgaaccccttatctgttttgtcaatttggactcctagatacttatttacctttcc
taatatttttaagttgaaatttttgcctagttgttcagcgaaattatcacatttaatttt
gtcgtgtccaaaatacaatatatcatcaacataaatcaaaacatattcaatagaatcatt
atttttttttgtgaaaaaacaaaaatcagatgaagtagctacaaaacccatatttttaag
tatctttgataagcactcataccaccgcttaccggactgttttagtccataaaggctctt
gtttaatagccaaatttctccctttttttgtatcctttcctgggggtgctttcacataaat
ttctttatctagatcagcgtgcagataggctgtctgcacgtcaaattgatatacttccag
atttaagatagaagctaacactagtgctgttttcacgctttctgatctaacggttgggga
ataagtgtcttcatagtcatccggaaatctttgggcaaagccttgagccaccaacctggc
tttgtataggaatttaccatcctctagctccttgattttatagatccacctagtgccaat
aggggatttctcagtgggcaggtctgttggtgtatatacatttagttctctcaaactgtc
catctctttttttcattgcttcctcccacttactttttttcttctaggggtagcttaagtat
gtcatcatatgtgttaggagtcaggtgtttagcttggatagacagtacgtaatcatctaa
tcttttaggaattatgcccctattggatctaatgggcctattctggggatgttcatcgct
atgcatatacttagcttctttttcggttaattgatctactttcaaagtggggtgtccttt
tttccattcatcattaactattactgggcatttgattctgttagtagatgtggatttggg
gttcgtatctgatgtgtgatgttttgcgtgcgtgtgtgtaatatttttttgattctgggta
aatttcattgagatgtaattttgaccagttcatgtgttcatgaaacacggcagatctgga
tacaatgaagtgattatttttatcaatgaagcgatatcccttggttcctgattcgtaccc
cataaatatcagtcttttggatactttctctcctttgcgtcttaactgtgagggcacata
tacatcagcagatacaccaaatacgtgtaaatgcccaattttttggtttccttccatacat
gagttcgtatggggtcttattgattgtactggaccataagcgatttatagtgtaattgga
ataattaagggcttcagcccagaatgactggtttaaaccagcatcatctaacacacattt
aaccatggtttgtagcaaaccattcttcctttcagagactccattttggaatggggaaaa
tggacaagatttaaagtgcaatattccttgtgattttagccaaattgaaaattcgttgga
taggaattccgacccccctgtcagtcattatttgtttgattttggtattgtattgtgtttc
taccattttaaagaactccttaaatttttttgaaaacctcacttttgtggttaagggtgta
tacaaaaccaaatctactatagtcatcgactatggtcaggaagtatctttttttttgtattt
aattgaaggttttaatgggcctactacatccatatgaattagttgaaaggttgattgtgt
ggtgcgggtggcttttttgggttttggcttgtttttaattttggaagaattgcatacaga
gcattctaagtaatttttacagttctttatttttaaatcttcggaacactctaacatttt
tcgtatgactggaaagctcgcgtgtcctagctttctgtggagtaagtgactacaattatc
gtgaatagtttttccattgtttaaattttcagacatattagtatcagtgtttgatgcatt

cttatcattattttcatcatggctatctgaatttggtttttgtttcttaattgatagtt
tgcatagactagttcgttatcttgtggcagtaggtatgttagtccatcatttgtaaatag
tttacctatgtttctgttgttcttagtcatgaatacctttccacctctgaaagtggtttc
tattgcattgtctaagcttaactttagggttgccaatatgttatttggaatttcgggcac
aaatagtacccccctctatttttcctatacctttatatgcgtgtctcctattcctctcac
taggactctattttggtttgctactttaatataatatttcttttcagtgaagttacttaa
tatttctctattatgagttgtatggcatgtggctcctgaatctatgagccaggtgttgag
tgaatcagatatcaaagtgttattatttcttattccatatagtgatcctttcttattttc
tctagggttatttctgtatgtttgtctttctctagagtgtggtgattctcttctcttata
cctgtaatctggggaggttctgtctaaaaagtgtattcttctatccttgtgtttatatga
gtcactgtatcctatattcttgggacagtctctactctgatgtctttcagatccacatgt
gagacattcatgtgggtatctgtctctggccctgaaatcagtccttctttgatttctacc
ttctctttgttgtgtatatcgctctcttctaagtctatcttcagtaataagcttattggt
aaggttttctaaagtaatttgctctatacttaggttatctattagtgtaatgtagttatc
ccagctgtgatctagtgagttaattataatgcttttcatggtgttgtcagaaatggtttc
tcccctttgaataaggtctgctcttacagcgtttaaattcaagatatgctcactcatgga
tgtatcatgttttagtttggcagaatagaattccctagagattttgtggtagtggctac
atttttgctatagaacagtctgaataatttttgatatacttcaaatacagttaaattatg
ctctatactggggagtatacttggggaaatagaaacaattaacatatagtatgctttgtg
gtggaggcagtctatttcataggtccaaggcttatttggtgggttatcaataaaaaataa
aacatttctttcatatatacaatctgtaaacactgatcccattccattaagttgcttcc
atctaatttcaattttataaagtctggaggtattgtccctgagtgtagtgaggtgttatt
catgtttagtagctgtccatgatttaagaccttagcatactcacaataaggcctgggtat
ggtagatgtatgtctgtatttacttatctctatgttgcgctgacttgtatttatgtttcc
ttcttctttctttgcagGCTCGTTCTGGGCATGCTCAGTCCTCATTTTTCTCCAGCAGTC
GATGAGGGAGGTTCCTGTGTCTGCAGCTCCTGGGGTGAAGTCCCAGACGGCCTTCTGTTG
CTCTGCTTTGTTTCCGGAGGGCCTCTGGTGACTGAAGAATGCCTGGGGTGCTGCTCTGCT
CATCGGGGAGTCTTCTCTGAGGTCGTCAGTAAGTGGAGCTTGAGGCGGGCCGCCATTGCC
AATGGAGCCGGGTTGTTCAGGGAGCTGCTCTTCAGTAGgtaagtatgattgccagcttgt
ctttcttcgggtggctaggcctttctttagacagtctatttctttttggcctttggtaga
tggtagtttagaattttctatttcattttctggttgtttgtttggtattgttagtttagt
cccgagtgctgcagttagcacctgtatagcattgtctatttctacttcagtaggttgagc
tttgttttgtttaaataattgtttttatttgtttttggactcgggtaatcatcttcttg
gtaggttttgtttttatttcgtagtttgaggtttggaattcttttagtaattggtcagg
cattttagaggtattaggtgggtaagatggctgccatgaaaaaggcgggaagctgtcgcc
atctttggtgtcatttttgaagtcaggggttgaatttcttaccttgcagttttcttgttt
gttattttcgttatctgctgctgttccgtggtcgtttattatgctgcggattttcacaag
cagatttgattcagctgtagttgtttctattaattctagtatttttctttttacctcatc
ttctttgtttggtgttagcagttcattttcttgtaattgcgtttgtttcttaatattttc
aagtggtgtatatttgtcaggagttagttgagctgccattcttttcgttatgtatctggt
ggcaggtcttcttttgtcgttatgtgagtcttgcatctttatgttcggttgggcctacca
aaccatcctcttgctaccaatttgttgctagtttggttatcctggttaggcccatatata
agagactgtaggctttcttgtaatagtaatatttatttcttttcttcctcagGAACACAA
TTAGACAGGCACTAATCTGGGAGGAGCAAGAACTTATATACAGAGAAGGCGGGGCTAATg

gtaagtactaaccaatggtaagtgacacaatgacatcacacaatgacatataaacaagca
cacatatttacagatatcaggttaggtattttcatctgtaacaatcccaaatatggcagg
gaactggtcacaaatatgtaccactagcctgccggtccctattttgggctctccaatccc
ttcaggctttttcaggtcagcccttttccagggtatcagggtgtaattatctccctgaga
gaaaatgtcttacctggggcaagtagttaaactgttaccacttggttttaggtgaagaaa
tgctgtttcttcccaatcctttctctgaatctggatgggaaagaacaggcaattactctt
ctcagtggcttaatttctaagcacctagctttgatttccctctctaaaacgatccaagat
ctttttagtttagctgttgagcaatgccaggtccagtcagaaaatctgtttctgtttcta
cactccttcttattctgaattctgctgccaataaagatattgctaactacctctatttgt
gagagcttttacagagatagactatttctacatcatctcctagttggcaggccactccat
tcacaactagaacgttactgaatttggatcagttcaaggatggctgtttctgagctagtt
gttgaaaaagagcaatcttcattctttcactatatactgtagatgagaatgtatgctgtg
agtccaagatcttgctttacatgcttatagtttctaaatgtcttgattttggcctggtgg
tatttctcattagagttaattattatgtataaatgaataaatctgctttgaaaaatctct
ccattaactaaagtgaaaaaaaatatcaacaaatggctatactttttctgcataaaacac
agttggatatgatttagttccatagtagctccctatgggaatataaaccagggaatcagc
agcattttcaccccagagtgctagataaagtgggagtgagagatactcaaatgtacatat
gtaggaatatgtagattttgacgacgaccccctggtaagtcaacatgtggtccctgtgac
catcctggtggaattagtggtgccctcattgggcacccaggggaggatgttggccctatt
ggttttgggttgcaccaggtgtatggttaggttgtagagaaattgggactgagactgtgg
agattgaggatgcccatagccgaatgacaattggatggattggtggtgggtgggtgggtg
gatgggtgaggaagctcacttcataatggaacttgtggaaatgaggatggggagacacac
agagtttctgagttttatagtagcacctgaaacatttactaagtgagtcctccttctatg
tcctgttttcctgaaaatgttcagtagtgtaagtgcatttgggtgaggcaaagggcagaa
ggaacacataattggctacacagaagtcttgaaatattagagaaataaaagtcactggta
gagtactaccaagttgcaagaatatagcattaatcccaaagttgcagcttctgcctccca
atggctagcaattttccttttaactggcataggaatgattcaaacctgtatcaatattgg
gtttatccaaaactgaaatactttcaaaagaaagatttaggaataatctactcgctccgt
gaccattcatatgttttaagatctttcattaaagtggatgcacattcctatccactctaa
aaaaagaagcccccaaataattgttttcaactctgaaaaagaatttgcactgaaaatctg
ctctgggattcatttaatgacattagttcactgaaggaaaatggcacgttcaatttcatc
tgaaagattctccaaaagagtcatatttacctatgtaaaacaaattcaaacatagcagca
cagctcttttaaaagatttctgtctttctAgATGCTTTTGGTTGCTATCTTCTCTGAAAC
TGGGTTTTTTGATTACTGTGCAGTAAAG
